# Low Temperature MOCVD Synthesis of High‐Mobility 2D InSe

**DOI:** 10.1002/smll.202510911

**Published:** 2026-01-25

**Authors:** Robin Günkel, Oliver Maßmeyer, Markus Stein, Kalle Bräumer, Rodrigo Sandoval Rodriguez, Daniel Anders, Jan‐Heinrich Littmann, Sebastian Anhäuser, Badrosadat Ojaghi Dogahe, Max Bergmann, Milan Solanki, Nils Fritjof Langlotz, Johannes Glowatzki, Jürgen Belz, Andreas Beyer, Gregor Witte, Sangam Chatterjee, Kerstin Volz

**Affiliations:** ^1^ mar.quest | Marburg Center for Quantum Materials and Sustainable Technologies Philipps‐ Universität Marburg Marburg Germany; ^2^ Department of Physics Philipps‐Universität Marburg Marburg Germany; ^3^ Institute of Experimental Physics I and Center for Materials Research Justus Liebig University Giessen Giessen Germany

**Keywords:** 2D materials, indium selenide, In_x_Se_y_ phase diagram, metal organic chemical vapor deposition

## Abstract

2D indium selenide (InSe) is a layered semiconductor with high electron mobility and a tunable band gap ranging from 1.25 eV in the bulk to 2.8 eV in the monolayer limit. However, growing phase‐pure InSe remains challenging due to the complex indium–selenium (In–Se) phase diagram. This complexity and the sensitivity of chemical precursors to growth conditions make it difficult to control which In–Se phase forms during synthesis during, e.g., metal‐organic chemical vapor deposition (MOCVD). MOCVD is considered the most promising approach for growing InSe, as it enables wafer‐scale, uniform, and controllable deposition—key requirements for device integration. We present a systematic investigation of InSe synthesis on c‐plane sapphire substrates at low temperatures. By varying Se/In precursor ratio and growth temperature, we create a phase diagram that covers In‐rich, equal stoichiometric, and Se‐rich In_x_Se_y_ phases. Raman spectroscopy and atomic force microscopy, supported by energy dispersive X‐ray spectroscopy and scanning transmission electron microscopy, reveal formation conditions of 2D InSe. The epitaxial alignment is verified by in‐plane X‐ray diffraction. Samples grown under optimized conditions exhibit a strong optical absorption in the visible range and especially a comparably high electron mobility, underlining the potential of the MOCVD‐grown material for future applications.

## Graphical abstract

We report on the low‐temperature, wafer‐scale metal‐organic chemical vapor deposition (MOCVD) growth of phase‐pure two‐dimensional indium selenide (InSe). By adjusting the Se/In ratio and temperature, we mapped In‐rich to Se‐rich In_x_Se_y_ phase space and identified the conditions that produce epitaxial, layered InSe. Structural and spectroscopic analyses confirm films with visible absorption and high electron mobility, highlighting the potential for applications.

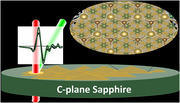

## 1 Introduction

The study of 2D materials has attracted considerable interest, particularly following the exfoliation of graphene and the studies of its electronic structure that led to the 2010 Nobel Prize in Physics [1, 2]. Graphene, along with other 2D materials, offers significant potential for further miniaturization of logic‐based devices [3, 4, 5]. However, for these materials to be suitable for future applications, they must not only exhibit superior properties compared to existing solutions, but also enable a scalable and cost‐efficient synthesis, compatible with current technology platforms [6].

Among 2D materials, indium selenide (InSe) stands out due to its high field effect mobility, making it a promising candidate for next‐generation logic devices [7, 8]. Prototype devices based on InSe have already been demonstrated [9, 10, 11, 12, 13, 14]. Furthermore, light matter interaction [15, 16, 17, 18], for example, the strongly layer‐tunable band gap from 1.25 eV in the bulk to 2.8 eV in the monolayer limit [14, 19, 20, 21] and potential optoelectronic applications [15] as for example photodetectors [22, 23, 24] or gas sensors [25, 26] based on InSe are investigated. For practical applications, InSe must be synthesized using scalable methods that ensure reproducibility [27, 28]. One challenge for the bottom‐up synthesis using different growth techniques is the large number of existing In‐Se phases [29, 30]. Nevertheless, first thin film deposition experiments demonstrate phase control, for example, via Molecular Beam Epitaxy (MBE) [31, 32, 33, 34, 35, 36, 37], Atomic Layer Deposition (ALD) [38], Physical Vapor Deposition (PVD) [39, 40] or Pulsed Laser Deposition (PLD) [41, 42]. Furthermore, Chemical Vapor Deposition (CVD) of InSe has previously been demonstrated on mica substrates at elevated temperatures of 600°C [43] and 630°C [44]. On c‐plane sapphire, metal‐organic chemical vapor deposition (MOCVD) of InSe using trimethylindium (TMIn) and diethyl selenium (DESe) has been reported within a process window of 500°C–600°C [45]. However, for compatibility with complementary metal‐oxide‐semiconductor (CMOS) technology, reduced thermal loads of ≤ 400°C are required to enable in‐line integration with back‐end‐of‐line (BEOL) processes [46, 47, 48, 49, 50, 51, 52, 53, 54]. Monolithic 3D integrated circuits (M3D‐ICs) show great promise for incorporating 2D materials as channel materials [55, 56, 57]. Below this BEOL compatible threshold temperature of 400°C, single‐source precursor approaches have been explored for In_x_Se_y_ deposition [58, 59]; however, these have yet to achieve the formation of layered 2D InSe. Recently, a modulated precursor supply strategy has enabled the growth of phase‐stable, layered InSe in the 350°C–500°C temperature range [13] with smooth 2D layers demonstrated at temperatures of 500°C and above. This first report on 2D InSe synthesis by MOCVD demonstrates its growth in a vertical showerhead reactor using TMIn and dimethyl selenide (DMSe) as precursor sources [13]. To reduce the deposition temperature of smooth layers further, Se‐precursors featuring a lower decomposition temperature compared to DMSe are important, as the effectiveness of MOCVD growth depends strongly on the activation energy for the thermal decomposition of the employed precursors, which determines the lower limit of their efficient use. Among the common organo‐selenium precursors — DMSe, DESe, and diisopropyl selenide (DiPSe) — DiPSe exhibits the lowest activation energy for thermal decomposition [60], making it particularly suitable for low‐temperature MOCVD processes. DiPSe has been successfully used for the MOCVD growth of several other chalcogenide materials, including Cu(GaIn)Se_2_ [61], 2D GaSe on Si(111) [62], and 2D WSe_2_ [63, 64], further underlining its versatility.

In this work, we focus on the controlled growth and comprehensive characterization of 2D InSe synthesized via MOCVD. While previous studies have successfully demonstrated the MOCVD synthesis of β‐In_2_Se_3_ [13, 65] and even realized ferroelectric transistors based on this phase [66], these efforts did not address the growth challenges or application potential of equal stoichiometric InSe. We go beyond these reports by demonstrating the phase‐selective and low‐temperature growth of 2D InSe films using TMIn and DiPSe – precursors well‐established in the epitaxy of III–V and II–VI semiconductors [67]. Our goal is to enable controlled low‐temperature growth of 2D InSe films compatible with CMOS requirements. Moreover, we aim at demonstrating the superior, application‐relevant properties of the 2D phase of InSe grown by MOCVD in this temperature window.

In this work, we first demonstrate how temperature and precursor ratio influence the formation of distinct In_x_Se_y_ phases, as identified by Raman spectroscopy. Atomic force microscopy (AFM) reveals the transition from isolated flakes to continuous layered films with increasing growth time. Subsequently, scanning transmission electron microscopy (STEM) and in‐plane X‐ray diffraction (XRD) confirm the epitaxial alignment of the InSe layers with the c‐plane sapphire substrate. Optical transmission measurements reveal well‐defined excitonic absorption features, while optical‐pump terahertz‐probe (OPTP) spectroscopy uncovers ultrafast photoconductivity dynamics and demonstrates a high carrier mobility in continuous InSe films.

## 2 Results and Discussion

**FIGURE 1:**
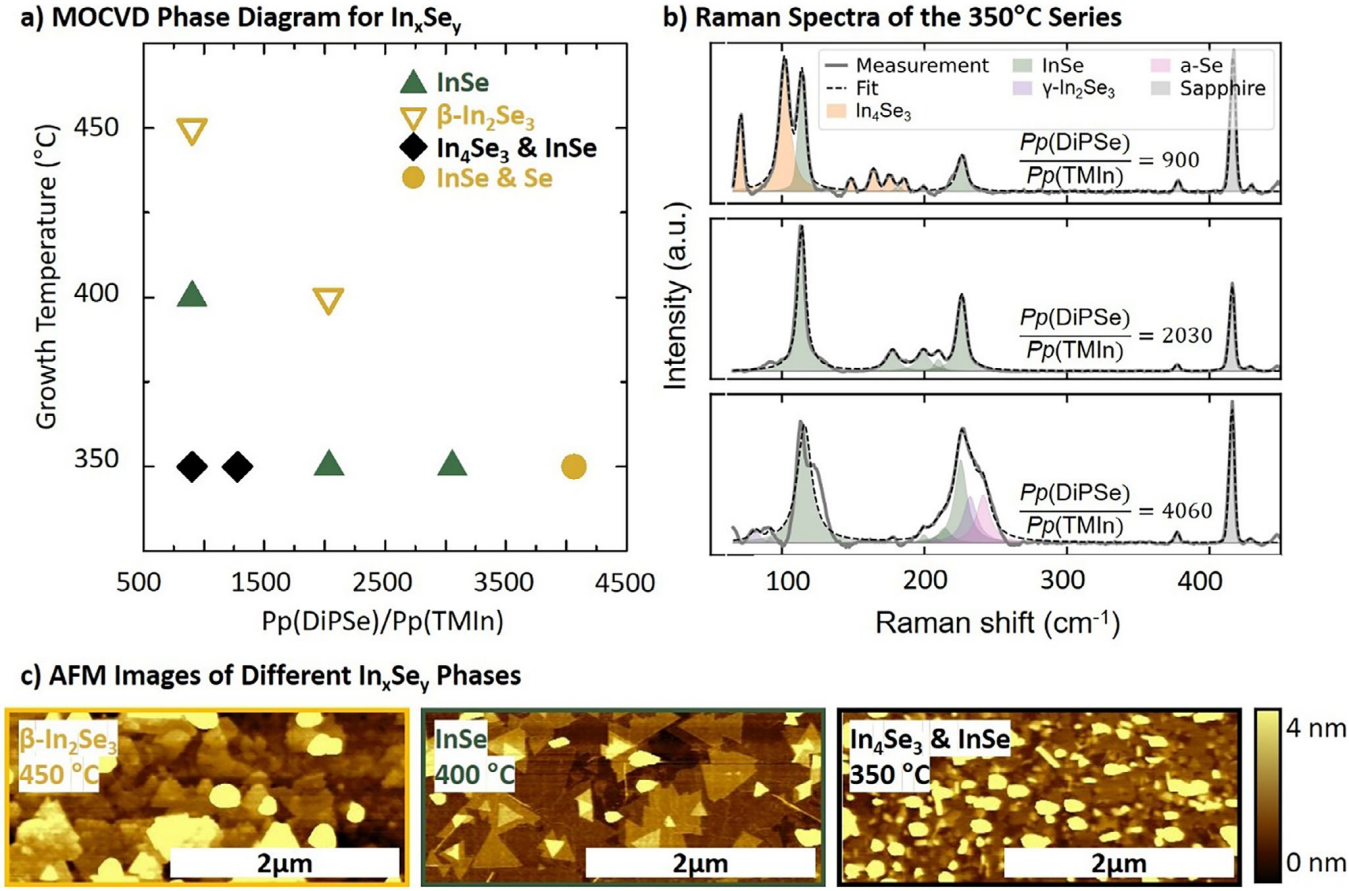
(a) Phase diagram showing the dominant In_x_Seᵧ phases as a function of the growth temperature (350°C–450°C) and the precursor ratio (900–4060). The targeted InSe is depicted as green triangles; Se‐rich phases appear in yellow, and In‐rich phases in black. b) Exemplary Raman spectra used to identify the different In_x_Seᵧ phases obtained at 350°C reactor temperature and a varied precursor ratio of Pp(DiPSe)/Pp(TMIn) = 900 up to 4060 (from top to bottom). c) AFM images illustrating the surface morphology of representative In_x_Seᵧ samples grown at a constant precursor ratio of 900 and temperatures of 450°C (yellow frame), 400°C (green frame), and 350°C (black frame), respectively.

Figure 1a presents the MOCVD phase diagram of In_x_Seᵧ as a function of the precursor ratio Pp(DiPSe)/Pp(TMIn) at constant TMIn supply and reactor temperature of 350°C, 400°C, and 450°C, respectively. Raman spectroscopy was used to identify the resulting phases by matching the measured spectra with reference data reported for InSe [37], β‐In_2_Se_3_ [37], γ‐In_2_Se_3_ [37], amorphous Selenide [68] and In_4_Se_3_ [69]. The corresponding AFM and Raman measurements for all samples are provided in Supporting Information Figures S1 and S2, respectively. As both the growth temperature and the selenium supply increase, the material transitions from In‐rich phases (e.g., In_4_Se_3_) to Se‐rich phases (e.g., β‐In_2_Se_3_ or γ‐In_2_Se_3_), with phase‐pure InSe forming under intermediate conditions. Figure 1b illustrates this effect in more detail by varying the DiPSe offer at a constant TMIn flux and at a constant temperature, resulting in distinct In_x_Seᵧ phases, each identified by their characteristic Raman signatures as referenced above. The emergence of specific phases is highly sensitive to the Se‐to‐In ratio. Notably, this study also demonstrates that phase‐pure InSe can be synthesized at temperatures as low as 350°C, as shown by the central spectrum of Figure 1b. Furthermore, the sample´s surface morphology correlates with the respective In_x_Seᵧ phase: the AFM analysis (Figure 1c) reveals that InSe forms well‐defined, layered triangular flakes approximately 1 µm in size, whereas mixed‐phase samples or samples mainly consisting of other In_x_Seᵧ phases show rougher surface morphologies. Before we now turn to investigate the InSe sample in more detail, we would like to emphasize that the nucleation conditions for a 2D‐material are very critical and hence, defined conditions need to be adjusted during the growth of the first layer. Figure S3 exemplarily shows the large influence of an Indium pre‐deposition step prior to In‐Se growth. As In will also be deposited from the reactor walls in the cleaning step of the substrate, we have consequently included an In pre‐deposition layer in all samples shown in the following, to have comparable starting conditions.

**FIGURE 2:**
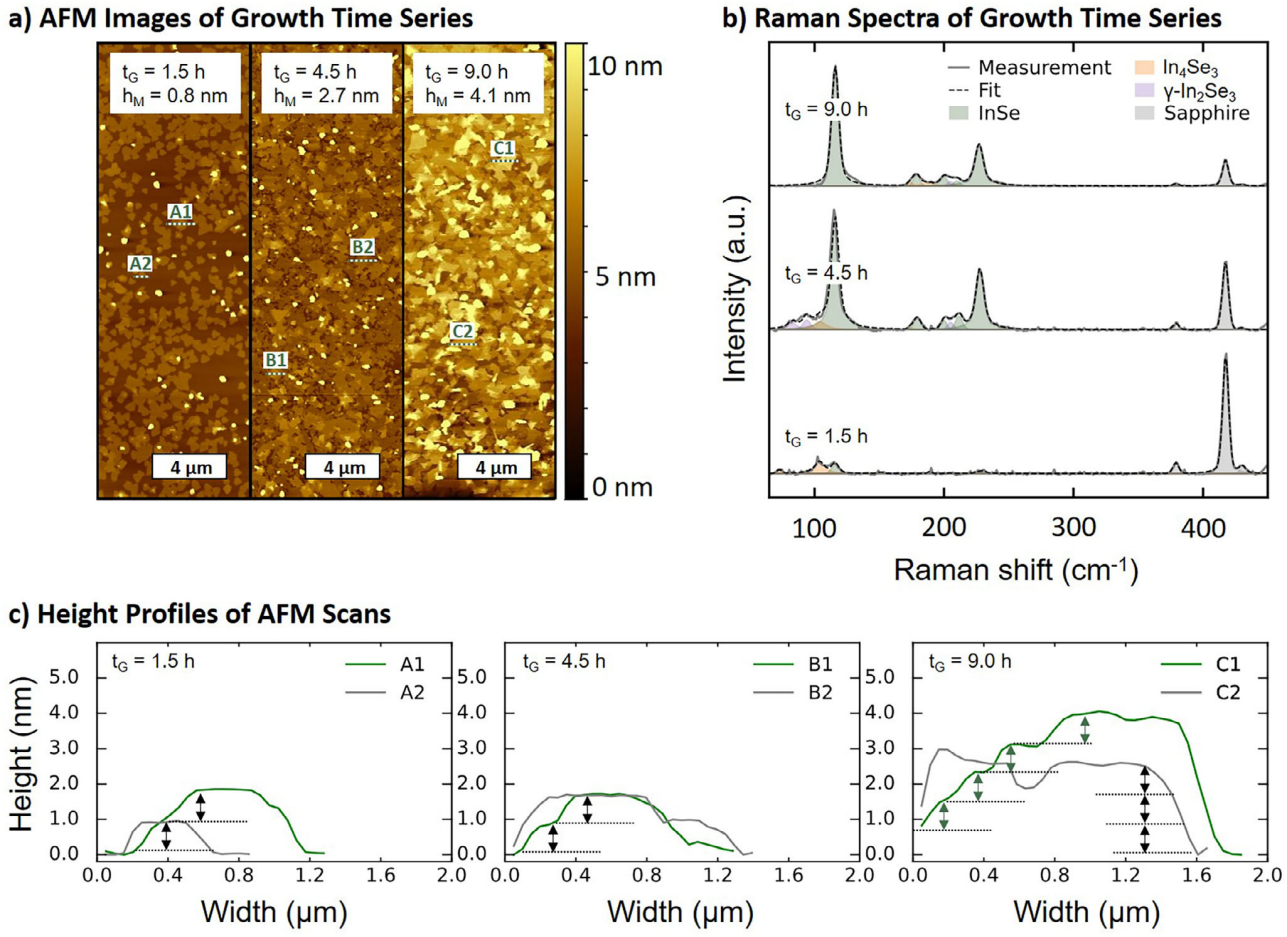
(a) Atomic force microscopy images of InSe samples grown for different durations: *t*_G_ = 1.0 h, 4.5 h, and 9.0 h, corresponding to median film heights of *h*_M_ = 0.8 nm, 2.7 nm, and 4.1 nm, respectively. b) Raman spectra showing the emergence of characteristic InSe vibrational modes, most pronounced in the 9 h sample (top box) and clearly visible in the 4.5 h sample (middle box), confirming the formation of the InSe phase. Compared to the sapphire background signal, the intensity of the InSe Raman modes increases significantly with longer growth duration, as seen from the 1.5 h sample (bottom box) to the 9 h sample. c) Three representative height profiles, from left to right, corresponding to samples grown for 1.5, 4.5, and 9 h, respectively.

**FIGURE 3:**
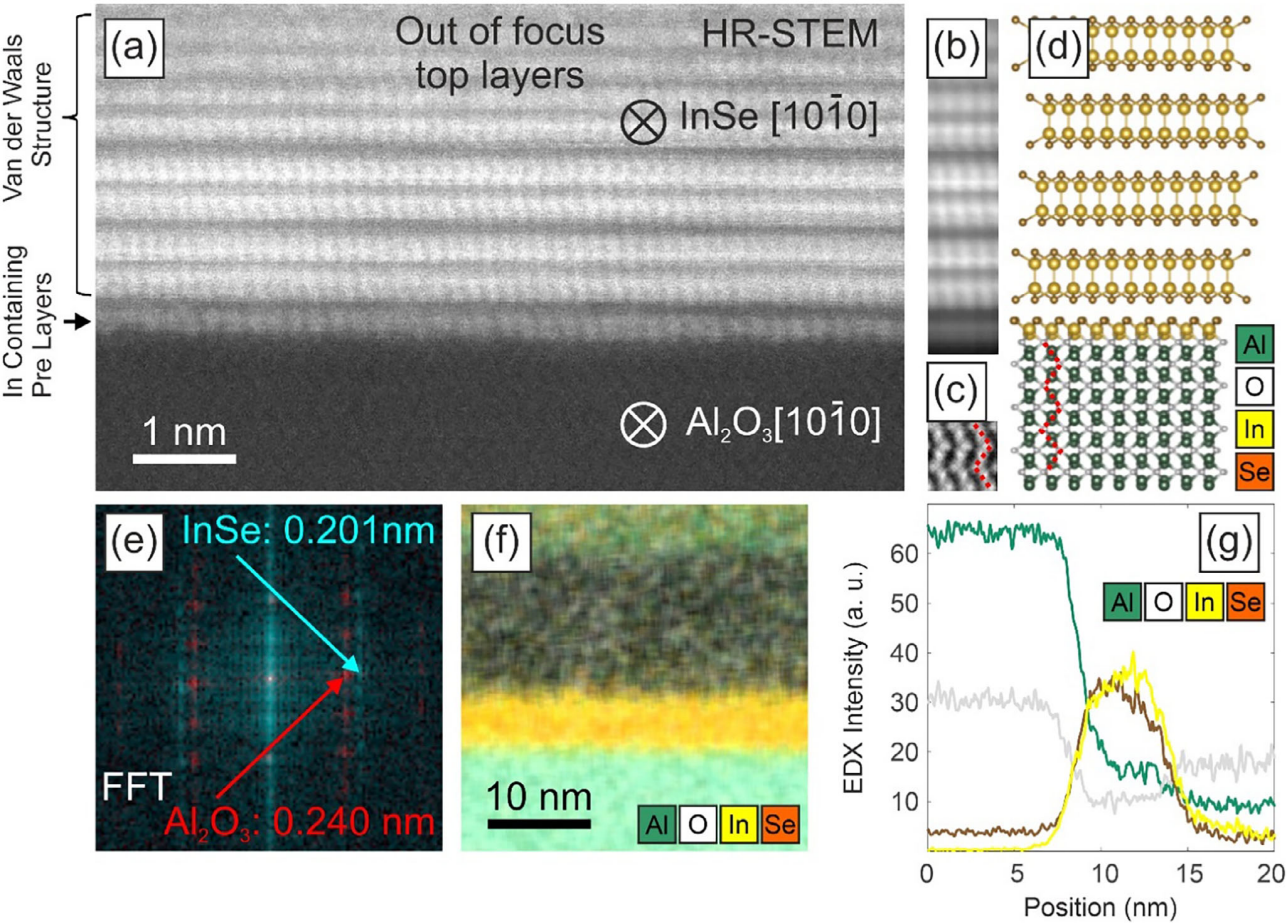
(a) Cross‐sectional scanning transmission electron microscopy (STEM) image of InSe (9 h growth time). b, c) Position‐averaged high‐resolution STEM image of InSe and sapphire, respectively. d) Ball‐and‐stick model for the InSe van‐der‐Waals‐like structure on sapphire. e) FFT of a) showing the periodicity of Al_2_O_3_ {11‐20} planes (red) and the InSe {11‐20} planes (cyan). f) EDX map of the InSe cross section showing the location of aluminum (green), oxygen (white), indium (yellow), and selenium (brown) using the Al‐Kα, O‐Kα, In‐Lα, and Se‐Kα lines, respectively. g) Line profile extracted from the EDX maps.

Figure 2a depicts an AFM height series of samples grown at 400°C with the optimized Se/In ratio to achieve single‐phase InSe, but for different times to investigate the morphology when the thickness is changing. As the growth time increases from 1.5 h to 9.0 h (from left to right), the median height *h_M_* of the height distribution in the AFM images concomitantly increases from 0.8 nm to 4.1 nm, corresponding to an increase from approximately one to five InSe layers, respectively. Furthermore, the line scans in Figure 2c reveal that the height profiles consist of multiples of approximately 0.8 nm, consistent with the monolayer thickness of InSe. Figure 2b displays the averaged and subsequently fitted Raman spectra corresponding to the samples. These fits consistently indicate the presence of the targeted InSe phase. However, the spectrum of the 1.5‐h sample shows additional Raman modes associated with In_4_Se_3_ [69], and the peak fit for the 4.5‐h sample includes features attributed to γ‐In_2_Se_3_ [37]. In contrast, the 9‐h sample exhibits a distinct Raman signature characteristic of phase‐pure InSe. The presence of secondary phases in the thinner films may serve as an initial indication of a covalently bonded interfacial layer on the sapphire surface and/or an incomplete nucleation process during the early stages of growth. We discuss this in context with the electron microscopy data of the interface between the substrate and the InSe (Figure 3). Calculating the difference between the A′₁(1) and A′₁(2) Raman mode positions of InSe, as motivated by Molas et al. [70], reveals a decrease in the difference with increasing growth time, i.e., increasing layer thickness. The difference in position decreases from 113 cm^−1^ to 112 cm^−1^ and finally to 111 cm^−1^ when increasing the InSe thickness from approximately 2 layers (expected height ∼1.6 nm) via 3–5 layers (expected height ∼2.4–4.0 nm) to 5–7 layers (expected height ∼4.0–5.6 nm), respectively. Our previously determined median heights of the three samples depicted in Figure 2 lie within these thickness ranges and also show the position of the Raman modes expected from literature, confirming the consistency between morphological and spectroscopic characterization. In addition, the AFM images (Figure S4a–c), out‐of‐plane XRD scan (Figure S4d), and corresponding radial Raman evaluation (Figure S4e) of the 9‐h‐grown sample across the wafer underline the homogeneous synthesis of InSe at wafer‐scale.

To further investigate the epitaxial alignment and interfacial structure between the InSe and the sapphire substrate, high‐resolution scanning transmission electron microscopy (HR‐STEM) is performed on cross‐sectional samples, as illustrated in Figure 3. The high‐resolution STEM cross‐section image (Figure 3a) confirms the identified 2D γ‐InSe phase. Due to an increased signal‐to‐noise ratio, the low electron dose that could be used on these samples, the crystal structure becomes more evident in the position‐averaged images of the InSe layers (Figure 3b) and the substrate region (Figure 3c). A ball‐and‐stick model depicting the arrangement of the hetero interface is depicted in Figure 3d. The crystallographic alignment between the sapphire substrate and the InSe layers is clearly visible from the FFT (Figure 3e). Here, the {11‐20} spots of the InSe are parallel to the {11‐20} spots of the sapphire. The lattice spacings are determined to be 0.201 nm and 0.240 nm for InSe and Al_2_O_3_, respectively. Both values are consistent with the expected lattice spacings of d_γ‐InSe_^{11‐20}^ = 0.201 nm and d_Al2O3_^{11‐20}^ = 0.240 nm, underlining unstrained growth of the van‐der‐Waals material on the substrate.

Next, we investigate the interface between the sapphire and the InSe in more detail: the c‐plane of pure sapphire is expected to be either aluminum or oxygen‐terminated [71]. However, the higher intensity observed in the HAADF image at the interface between the first Se layer of the 2D InSe and the last oxygen layer of the sapphire substrate (Figure 3a,b) suggests the presence of an initial nucleation layer containing In and/or Se, probably covalently bonded to the substrate. This is expected from the TMIn seeding approach and the following Se supply outlined in Figure S3. In‐containing interlayers have also been observed in MBE experiments [36]. An analogue study on GaS grown on sapphire reported a comparable Ga layer bound to the surface [72]. To further confirm the 1:1 stoichiometry of the InSe layers, complementary energy dispersive X‐ray spectroscopy (EDX) measurements were carried out. A map of the hetero interface is shown in Figure 3f. The individual concentration profiles derived from this map are shown in Figure 3g. Indeed, an equal concentration of In and Se is found within the InSe layers, reflecting the 1:1 stoichiometry of the γ‐InSe phase. This interfacial In_x_Seᵧ layer, which we observe at the interface, might well explain the small Raman peaks of non‐InSe‐phases for very thin layers, as concluded from Figure 2. It should also be noted that due to re‐absorption of the low‐energy X‐rays within the comparably thick TEM sample, the Al and O profiles can only be interpreted qualitatively.

**FIGURE 4:**
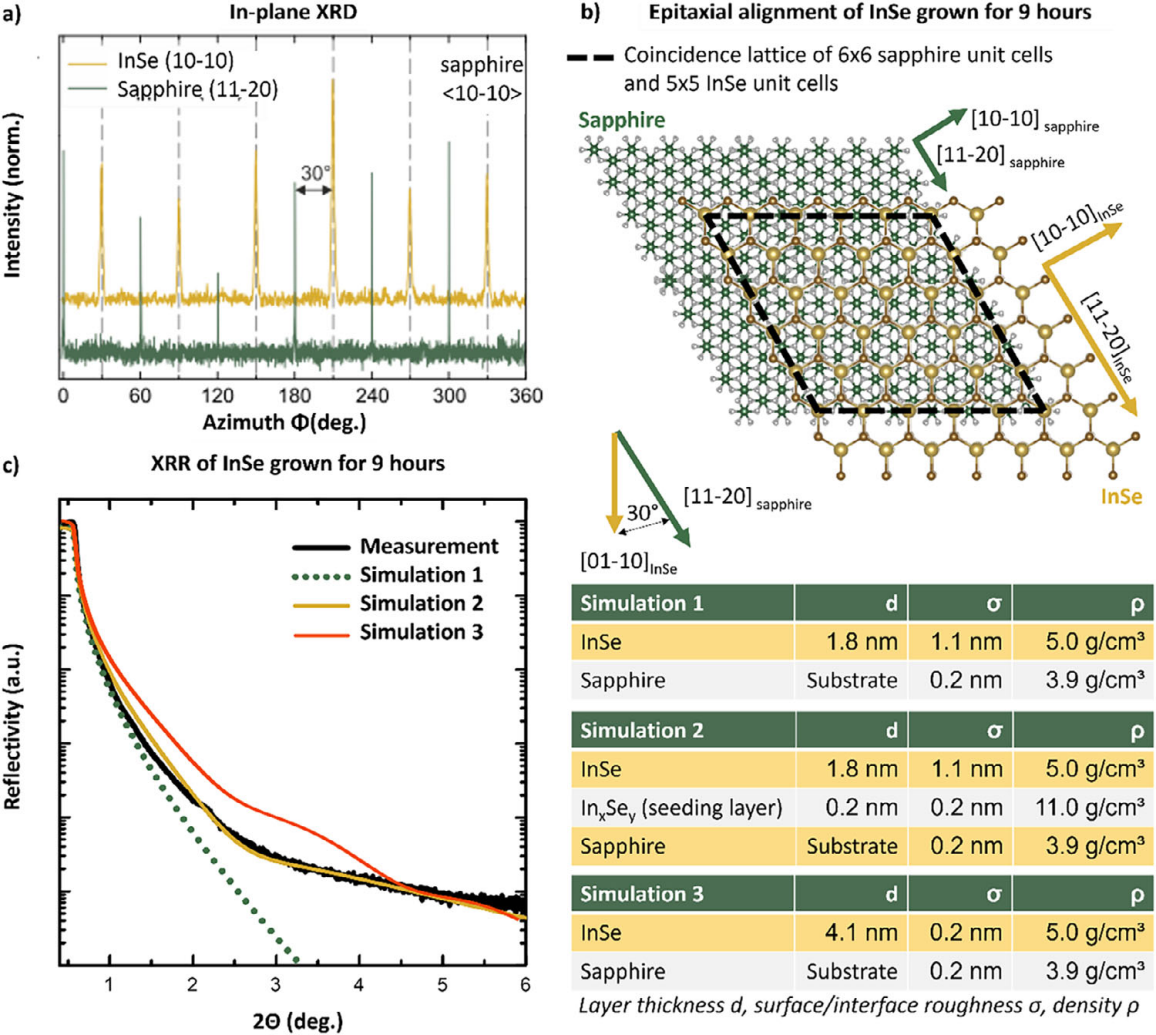
(a) In‐plane XRD Φ‐scan of {11‐20} reflections of sapphire (green) and of {10‐10} reflections of InSe (yellow), respectively. The corresponding azimuthal alignment of InSe to sapphire is shown by a top view model of the lattice planes in b). The coincidence lattice is shown in black, where 6 × 6 sapphire unit cells align with 5 × 5 InSe unit cells. c) The X‐ray reflection (XRR) measurement is plotted alongside the corresponding simulations: simulation 1 (InSe: 1.8 nm, σ = 1.1 nm, ρ = 5 g/cm^3^; sapphire: σ = 0.2 nm, ρ = 3.9 g/cm^3^), simulation 2 with the additional In_x_Se_y_ nucleation layer (InSe: 1.8 nm, σ = 1.1 nm, ρ = 5 g/cm^3^; In_x_Se_y_ layer: 0.2 nm, σ = 0.2 nm, ρ = 11.0 g/cm^3^; sapphire: σ = 0.2 nm, ρ = 3.9 g/cm^3^) and simulation 3 based on AFM measurements (InSe: 4.1 nm, σ = 0.2 nm, ρ = 5 g/cm^3^; sapphire: σ = 0.2 nm, ρ = 3.9 g/cm^3^). These simulations consider the layer thickness d, surface/interface roughness σ, and density ρ.

To verify that the azimuthal InSe orientation relative to the sapphire substrate is the same at the wafer scale as initially observed in the local STEM cross‐section analysis, we perform in‐plane XRD measurements of the 9‐h‐grown sample (Figure 4a). The angle between the sapphire {11‐20} plane and the InSe {10‐10} plane is determined to be 30°, meaning that the [10‐10] vectors of both materials are parallel. These findings confirm that the InSe, which exhibited a local alignment to the sapphire substrate as revealed by STEM analysis, maintains a consistent epitaxial relationship with the Al_2_O_3_ surface across the wafer. This alignment is illustrated by the schematic model in Figure 4b and corresponds to a calculated coincidence lattice, where 6 × 6 sapphire unit cells align with 5 × 5 InSe unit cells. X‐ray reflectivity (XRR) analysis (Figure 4c) is performed to verify the total thickness of the layered InSe crystal as well as to confirm the interfacial In_x_Seᵧ layer, as a structural continuation of the sapphire substrate, on a larger lateral scale than accessible by STEM. As a result of the STEM investigations (Figure 3f), two simulations, one without an In_x_Seᵧ interface layer on the sapphire (simulation 1), and a second one with an In_x_Seᵧ interface layer on the sapphire (simulation 2), were performed using X‐ray Calc3 [73]. Furthermore, a third simulation was performed based on the median height of AFM measurement (cf. Figure 2a). Simulation 2 confirms the presence of an In‐containing interface on the sapphire that forms a layer between the sapphire substrate and InSe, as seen in the STEM cross‐sectional image, supporting the expected growth process. As shown in the AFM image (Figure 2a), the amount of InSe layers locally varies by about 4 layers, resulting in lower material density on average for thicker layers, which causes a rough surface effect in the XRR signal, limiting the significance of the thickness evaluation based on XRR [74]. Furthermore, the simulated InSe layer thickness of d = 1.8 nm differs from the median height of h_M_ = 4.1 nm determined from the corresponding AFM images. This can be partially explained by the XRR simulated surface roughness of σ = 1.1 nm. However, simulation 3 reproduces the hump at an angle of 2°, which is also visible in the measurement. Because the X‐ray spot is large in relation to the AFM measurement, the XRR signal is a mixture of several InSe flake layers, preventing distinct Kiessig fringes from occurring. Finally, the XRR measurement supported by simulation 2 shows that there is an In_x_Seᵧ interlayer between the sapphire substrate and the 2D InSe on a wafer scale, as expected from the growth process (TMIn seeding) and already observed by STEM EDX and HAADF analysis (Figure 3).

**FIGURE 5:**
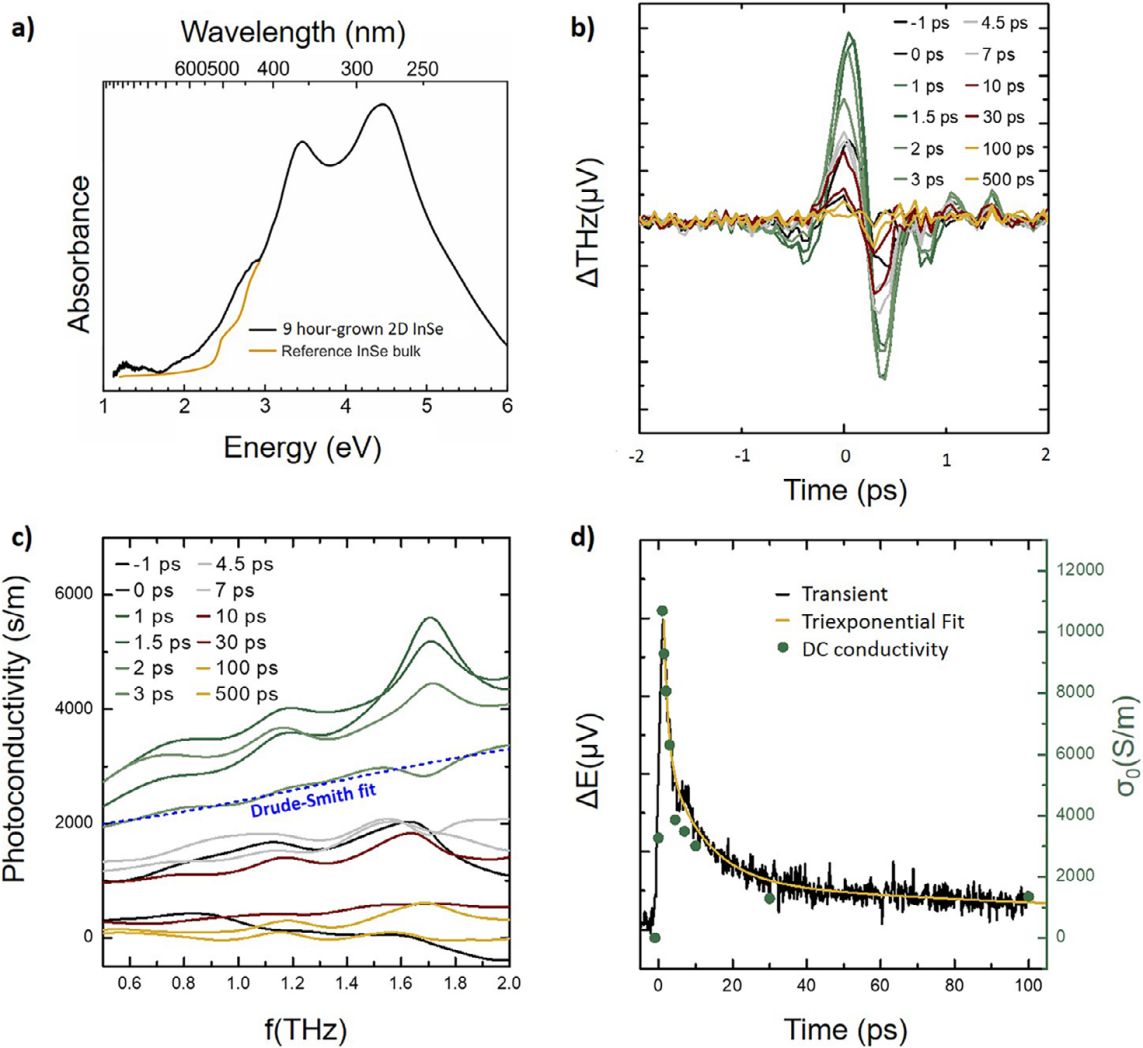
(a) Optical absorption spectrum of InSe grown for 9 h on c‐plane double‐side polished sapphire at a growth temperature of 400°C measured in transmission geometry (black). Reference measurement (yellow) of bulk InSe adapted from Segura et al. [75]. b) Differential THz time‐domain traces obtained by subtracting the THz waveform of the optically excited sample from that of the unexcited sample, shown for various temporal delays between optical pump and THz‐probe. c) Real part of the extracted photoconductivity in the THz frequency range for the different temporal delays, together with an exemplary fit of the Drude–Smith model. d) Transient signal at the peak of the differential THz trace (black line), alongside the extracted DC conductivity from the Drude–Smith fits (green spheres). A tri‐exponential fit (yellow line) is used to determine the decay dynamics of the light‐induced photoconductivity.

To assess the material's technological applicability, we perform a comprehensive functional characterization of the InSe films, as shown in Figure 5. Optical transmission measurements provide information relevant for photonic applications. Finally, terahertz (THz) time‐domain spectroscopy following optical excitation enables the extraction of charge carrier mobility, a critical figure of merit for electronic device performance.

To investigate the optical properties of MOCVD‐grown InSe, absorption measurements are conducted in the range of 1 eV to 6 eV using a transmission geometry. For these measurements, the InSe sample was grown on double‐side‐polished c‐plane sapphire with a layer number or thickness similar to that of the 9‐h growth sample (as shown in AFM data in Figure 2a; Figure S4). Its optical absorption spectrum is shown in Figure 5a in black, which is compared with a reference bulk measurement by Segura et al. [75] (in yellow). For comparability, the reference data is scaled to the absorbance of our measurement at 1.2 eV. The peaks at 2.9, 3.2, and 4.2 eV are consistent with ab initio calculations that combine many‐body perturbation theory methods, such as the GW method, and the Bethe‐Salpeter equation [76]. It can be seen that the three peaks correspond to calculated excitations in the plane of the InSe, which fits very well with the geometrical conditions of the transmission experiment, since the light here is incident parallel to the normal vector (in the c direction). Additionally, a microscopic absorbance measurement on the sample piece, subsequently evaluated with OPTP measurements, revealed a band edge onset at Eg = 2.14 eV (Figure S6). Consequently, the InSe exhibits the expected optical absorption properties, which pave the way for device applications of MOCVD‐grown InSe.

Once the InSe flakes coalesce into a continuous layer, OPTP measurements reveal a pronounced transient photoconductivity response. The strong photoinduced change in the transmitted THz field confirms the generation of mobile charge carriers upon optical excitation, with the signal evolving systematically as a function of pump–probe delay (Figure 5b). The frequency‐dependent photoconductivity spectra are analyzed using the Drude–Smith model, which accounts for partially localized carrier transport due to backscattering (Figure 5c). From this analysis, we extract an intrinsic electron mobility of about 1200 cm^2^/Vs, assuming an effective electron mass of 0.1 times the mass of a free electron [77]. This value is in excellent agreement with reported mobilities for bulk InSe [7], suggesting that the favorable transport properties of the material are largely preserved during the MOCVD growth of few‐layer thick films. However, the substantial backscattering indicated by the Drude–Smith coefficient c_1_ of about ‐0.73, consistent with scattering at grain boundaries, interfaces, impurities, and defect states, reduces the intrinsic mobility to an effective value of about 325 cm^2^/Vs. This demonstrates that, although some degree of carrier localization is present, the favorable transport properties of InSe are largely preserved in these MOCVD‐grown few‐layer films, and the effective mobility still exceeds that of most monolayer TMDCs. The temporal evolution of the photoconductivity follows a multi‐exponential decay after excitation (cf. Figure 5d). An initial ultrafast decay with a time constant of about 1 ps is followed by a slower component on the order of 10 ps. A residual photoconductivity persists with a much slower decay time in the range of 200 ps, likely due to recombination from trap states or defects, and is subject to further investigation. Immediately after photoexcitation, the carrier density peaks at 3*10^18^ cm^−3^ and subsequently decays according to these time constants. The momentum scattering time extracted from the Drude–Smith analysis is approximately 70 fs and remains essentially independent of both the excited carrier density and the pump–probe delay, indicating that the dominant scattering mechanisms are intrinsic to the grown InSe layers rather than governed by carrier–carrier interactions or transient excitation conditions.

## 3 Conclusion

This study demonstrates the successful synthesis of phase‐pure films of the 2D material InSe on c‐plane sapphire substrates using a horizontal metal‐organic chemical vapor deposition (MOCVD) system. Trimethylindium (TMIn) and diisopropyl selenide (DiPSe) serve as precursors at technologically relevant growth temperatures between 350°C and 450°C. A pre‐deposition of TMIn is employed to establish a reproducible indium‐based seeding layer. By optimizing the DiPSe‐to‐TMIn precursor ratio, phase purity is achieved, resulting in the exclusive formation of InSe at temperatures as low as 350°C–400°C. Raman spectroscopy confirms – for optimized growth conditions ‐ the InSe phase, with characteristic vibrational modes indicative of few‐layer, high‐quality material. Growth time series studied via atomic force microscopy (AFM) show the evolution from isolated triangular InSe flakes to continuous multilayer films. The epitaxial alignment and high crystallinity of the InSe films on sapphire are proven by high‐resolution scanning transmission electron microscopy (HR‐STEM) and in‐plane X‐ray diffraction (XRD), also on a wafer‐scale. Furthermore, a buried In_x_Se_y_ layer is identified via STEM‐based energy dispersive X‐ray spectroscopy (EDX) and X‐ray reflectivity (XRR) analysis. To validate the material's electronic and structural properties, optical absorption and optical‐pump terahertz‐probe (OPTP) spectroscopy were performed. THz measurements indicate electron mobilities to an effective value of 325 cm^2^/Vs, an essential parameter for high‐performance logic applications.

In summary, the controllable low‐temperature MOCVD process and the received mobility underline the potential for scalable and industrial integration of InSe into next‐generation logic devices.

## 4 Methods

### 4.1 MOCVD Growth and Sample Characterization

In this work, all growth experiments were conducted using a horizontal‐flow **metal‐organic chemical vapor deposition (MOCVD)** reactor (*Aixtron AIX 200 GFR*) equipped with gas foil rotation [78, 79], a platform that has previously enabled the successful growth of various indium‐based compound semiconductors, including InN [80], GaInAs [81, 82, 83] or GaInNAs [84, 85]. Infrared heating was used to heat the graphite susceptor. The reactor pressure was maintained at 50 mbar, and hydrogen (H_2_) was used as the carrier gas with a total flow rate of 6800 sccm for all experiments.

Moreover, we aim at precisely controlling the interface between the substrate and the 2D material, which is especially critical for the deposition of van der Waals materials, as they are – in contrast to the growth of covalently bonded crystals – grown without a buffer layer. Thereto, we introduce an In‐rich seeding layer prior to the InSe growth. Prior studies on the oriented growth of 2D WS_2_ on c‐plane sapphire have shown the importance of a controlled interface reconstruction: here, a tungsten‐based seeding layer was shown to promote a well‐ordered interface and epitaxial alignment of the subsequent 2D layers [86]. In our own previous work on GaS, a structurally similar post‐transition metal dichalcogenide [87], we also observed a gallium‐rich interfacial layer at the sapphire–GaS interface [72], which played a critical role in templating the layered growth.

The synthesis was performed in a temperature range from 350°C up to 450°C using diisopropyl selenium (DiPSe) and tri‐methyl indium (TMIn) as selenium and indium precursors, respectively. The DiPSe partial pressure was between 930 and 4060 times larger than the TMIn partial pressures. The ratio of the supplied precursors significantly exceeds the targeted incorporation ratio, which can be attributed to surface‐reaction‐limited growth kinetics. This behavior is well known from ZnSe synthesis using DiPSe below 440°C [60]. Prior to growth, the c‐plane sapphire substrate was preheated at 600°C for 22 min in order to reduce hydroxyl (─OH) group coverage on the surface and thereby improve surface cleanliness and reproducibility of the growth conditions [88]. After lowering the temperature to 400°C, the indium precursor was introduced for 170 s (TMIn predeposition, In seeding step) to promote the interaction between indium and the sapphire surface, thereby facilitating the catalytic decomposition of the selenium precursor introduced during the growth phase. This step ensures that selenium incorporation occurs from the onset of growth. After the growth time *t_G,_* the precursor flow was stopped, and the reactor was cooled to room temperature.

**Atomic force microscopy (AFM)** was used to analyze the sample topography using a Nanosurf Flex AFM system operated in dynamic force mode (tapping mode). Gwyddion software was used for data analysis and processing. Commercial probes from BudgetSensors Innovative Solutions Bulgaria Ltd. with a nominal tip radius of 10 nm were used for AFM measurements.

**Raman spectroscopy** was performed in backscattering geometry using a confocal Raman microscope (Horiba XPloRA Plus) with a 100×/0.8 NA objective and an excitation wavelength of 532 nm. After averaging Raman spectra collected over at least 10 × 10 spots with a spatial step size of ≥ 2 µm, background subtraction was performed using an asymmetrically reweighted penalized least squares smoothing [89]. To identify the present In_x_Seᵧ phases, the spectra were fitted using a pseudo‐Voigt function. The Lorentzian half‐width at half‐maximum (γ) was set to 5 cm^−1^, with allowed variation between 4 and 6 cm^−1^. The mixing parameter η, which defines the weighting between Gaussian (η = 0) and Lorentzian (η = 1) contributions, was fixed at 0.5 but permitted to vary within this full range to optimize the fit. Peak positions were initialized based on literature values for InSe [37], β‐In_2_Se_3_ [37], γ‐In_2_Se_3_ [37], amorphous Selenide [68], and In_4_Se_3_ [69], allowing for a deviation of ±3 cm^−1^ to accommodate experimental and material‐related factors.

**X‐ray diffraction (XRD)** was used to characterize the crystalline structure and identify film phases using a Bruker D8 Discover diffractometer with monochromated Cu‐Kα radiation and a LynxEye silicon strip detector.

**X‐ray reflectivity (XRR)** measurements were performed using a Panalytical X'Pert Pro diffractometer using Cu‐Kα radiation. The reflectivity data were analyzed using X‐ray Calc3 [73] software to extract the thickness and density of the InSe layer.

**Optical absorption spectra** were acquired in transmission geometry (spot size ∼ 3 mm diameter) using an OceanOptics HDX‐XR spectrometer (spectral resolution Δλ = 1.1 nm) together with an OceanOptics DH‐2000 combined halogen and deuterium light source. This enables both illumination and detection of light in the range from 205 to 1100 nm (1.13–6 eV).

### 4.2 TEM Sample Preparation

The TEM lamella was prepared using a Thermofisher Scientific Helios 5 Hydra CX, an analytical dual‐beam SEM / Plasma Focused Ion Beam (PFIB) instrument. Before FIB preparation, the sample was sputter‐coated with carbon prior to loading into the SEM. The initial pre‐coating was necessary to prevent the InSe layer from amorphization under electron beam exposure and to overcome charging issues since the substrate was sapphire (cf. Figure S5). The selected region of the sample was additionally protected with ≈ 1 µm thin carbon and tungsten layers, respectively. This procedure ensures sufficient surface protection from the xenon ion milling and subsequent fine polishing with argon ions.

### 4.3 STEM Measurement

Energy dispersive X‐ray (EDX) analysis and high‐resolution HAADF STEM imaging were performed in a double Cs‐corrected JEOL JEM‐2200FS operated at 200 kV acceleration voltage equipped with a Bruker Flash 5060 EDX detector. The quantitative elemental evaluation is based on the simulated Cliff‐Lorimer Methods [90] implemented in the Bruker Esprit 2.3 software. Rapid amorphization of the InSe layer was observed under the electron beam. To image the initial structure of the InSe layer, the imaging conditions were modified by reducing the sampling and dwell time per pixel of the STEM image. This resulted in a reduction of the electron dose by almost one order of magnitude from 2.8*10^5^ to 4.9*10^4^ e/A^2^, which significantly improved the imaging of the InSe layer.

### 4.4 Terahertz (THz) Transmission Spectroscopy

The OPTP setup utilizes a 5 kHz regenerative amplifier (Spectra Physics Solstice Ace) delivering 50 fs pulses spectrally centered around 800 nm. The output is split into three parts. The first part excites a large‐aperture LT‐grown GaAs antenna, which generates ∼1 ps long terahertz (THz) pulses. These pulses are then transmitted through the InSe samples to probe their photoinduced conductivity. The second part serves as the gate pulse for electro‐optic sampling, using a 500 µm thick ZnTe crystal cut along the ⟨110⟩ orientation. This enables the measurement of the THz electric field in the time domain. The third part of the 800 nm output is directed into an optical parametric amplifier (OPA, Spectra Physics TOPAS) to generate pump pulses with a central photon energy of 2.6 eV (480 nm). The pump pulse is temporally delayed relative to the THz probe by a motorized linear translation stage and modulated by an optical chopper before exciting the InSe sample. A schematic illustration of the experimental setup is shown in Figure S7.

The setup enables the detection of THz pulses covering a bandwidth from 0.3 to 2.3 THz, which are resolved in the time domain by electro‐optic sampling. We record a time window of 9 ps, to which we apply a Blackman–Nuttall window with a 2 ps slope prior to Fourier transformation. This procedure yields the complex reference spectrum E(ω) as well as the pump‐induced change ΔE(ω) caused by the modulated pump excitation.

The corresponding change in optical conductivity Δσ(ω) is then calculated as:

(1) $$\Delta \sigma \left(\omega \right)=\left(2c_{0}\varepsilon_{0}\sqrt{\varepsilon_{r}}\right)\;/L\cdot \left(\Delta E\left(\omega \right)\;/E\left(\omega \right)\right)$$

Where c_0_ is the speed of light in vacuum, ε_0_ is the vacuum permittivity, ε_r_ is the relative dielectric constant of the sample, and L is the effective thickness of the photoexcited layer. The resulting frequency‐dependent photoconductivity is analyzed by fitting the Drude‐Smith model, which is given by:

(2) $$\sigma \left(\omega \right)=\left[ne^{2}\tau /m*\right]\times \left[1/\;\left(1-i\omega \tau \right)\right]\times \left[1+c_{1}/\;\left(1-i\omega \tau \right)\right]$$

Where n is the carrier density, e is the elementary charge, τ is the carrier scattering time, m* is the effective mass, and c₁ is the persistence of velocity parameter describing carrier backscattering.

## Conflicts of Interest

The authors declare no conflicts of interest.

## Supporting information




**Supporting File**: smll72193‐sup‐0001‐SuppMat.pdf

## Data Availability

The data that support the findings of this study are available from the corresponding author upon reasonable request.
