# Supplementary material for: Low Temperature MOCVD Synthesis of High‐Mobility 2D InSe
Source: Small. 2026 Jan 25;22(11):e10911. doi: 10.1002/smll.202510911 (PMC12921547; doi:10.1002/smll.202510911)
Supplement: Supplementary file 1 — Supporting File: smll72193‐sup‐0001‐SuppMat.pdf [file SMLL-22-e10911-s001.pdf]

Supporting Information

Low Temperature MOCVD Synthesis of high-mobility 2D InSe

Robin Günkel[1,2], Oliver Maßmeyer[1,2], Markus Stein[3], Kalle Bräumer[3], Rodrigo Sandoval Rodriguez[3], Daniel Anders[3], Jan-Heinrich Littmann[3], Sebastian Anhäuser[1,2], Badrosadat Ojaghi Dogahe[1,2], Max Bergmann[1,2], Milan Solanki[1,2], Nils Fritjof Langlotz[1,2], Johannes Glowatzki[1,2], Jürgen Belz[1,2], Andreas Beyer[1,2], Gregor Witte[1,2], Sangam Chatterjee[3], Kerstin Volz[1,2],

[1]mar.quest | Marburg Center for Quantum Materials and Sustainable Technologies, Philipps-Universität Marburg, 35032 Marburg, Germany
[2]Department of Physics, Philipps-Universität Marburg, 35032 Marburg, Germany
[3]Institute of Experimental Physics I and Center for Materials Research, Justus Liebig University Giessen, Germany

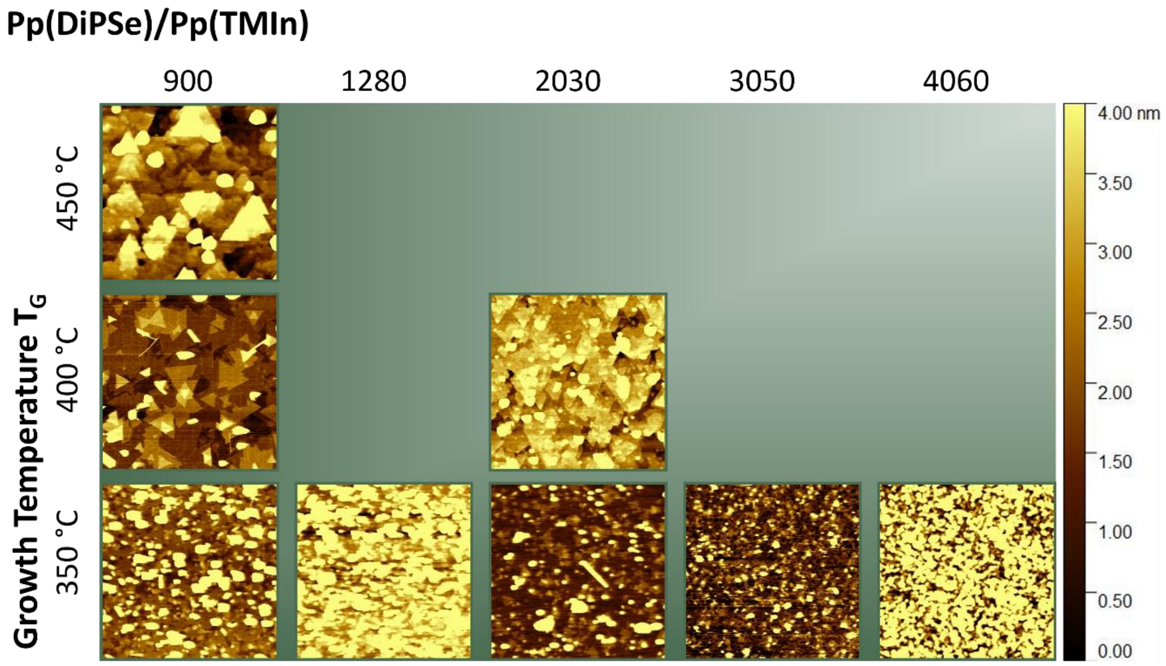

Figure SI1: Surface morphologies of In_xSe_y layers in dependence on growth conditions: AFM images of various In_xSe_y layers in dependence on growth conditions arranged in accordance with the phase diagram (Figure 1) to illustrate, how the surface morphology evolves with the observed In_xSe_y phases, depending on the growth temperature (350 °C to 450 °C) and precursor ratio (900 to 4060).

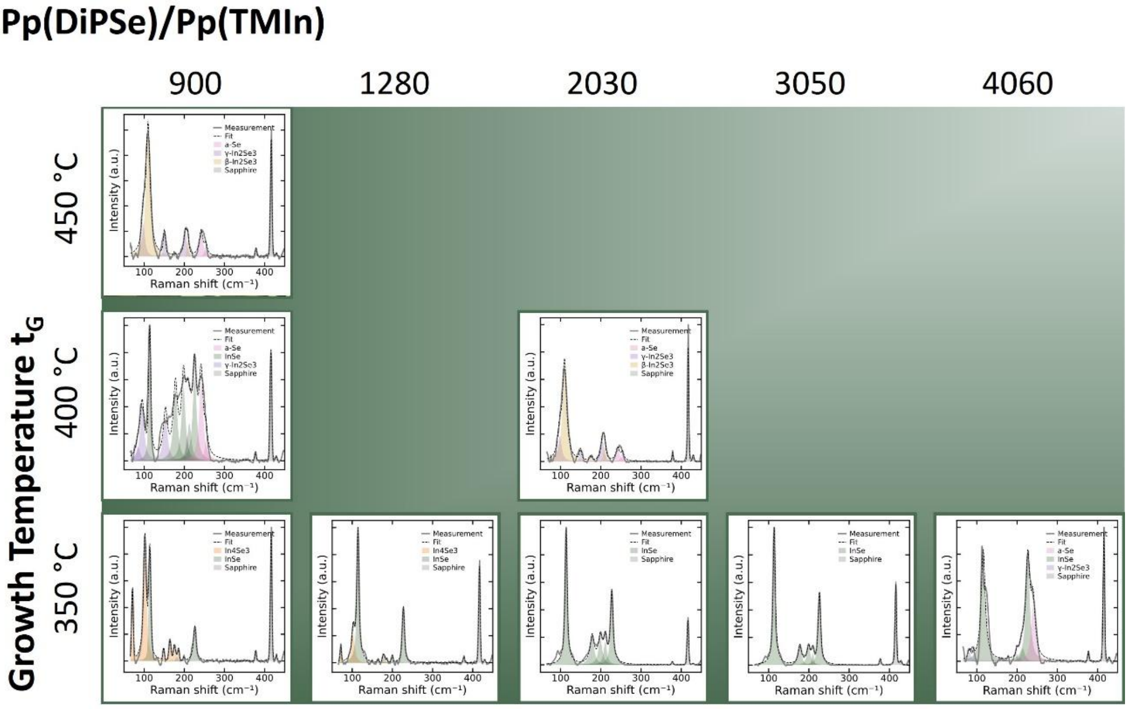

Figure SI2: Phase compositon of In_xSe_y layers in dependence on growth conditions: Fitted Raman spectra arranged analogously to the AFM images, following the phase diagram as a function of growth temperature (350 °C to 450 °C) and precursor ratio (Pp(DiPSe)/Pp(TMIn) from 900 to 4060). Spectral evaluation is based on spatially averaged Raman data acquired from at least 10 × 10 measurement points with a step size ≥ 2 µm. The background subtraction is performed using asymmetrically re-weighted penalized least squares smoothing[1]. Phase identification is carried out by fitting the spectra with pseudo-Voigt profiles, using a Lorentzian half-width at half-maximum (γ) of 5 cm^{-1} (allowed range: 4–6 cm^{-1}). The mixing parameter η, indicating the ratio of Gaussian (η = 0) to Lorentzian (η = 1) contributions, is initially fixed at 0.5 and permitted to vary during fitting. Initial peak positions are taken from literature for InSe[2], β-In_2Se_3[2], γ-In_2Se_3[2], amorphous Se[3], and In_4Se_3[4], with a tolerance of ±3 cm^{-1} to accommodate experimental variability.

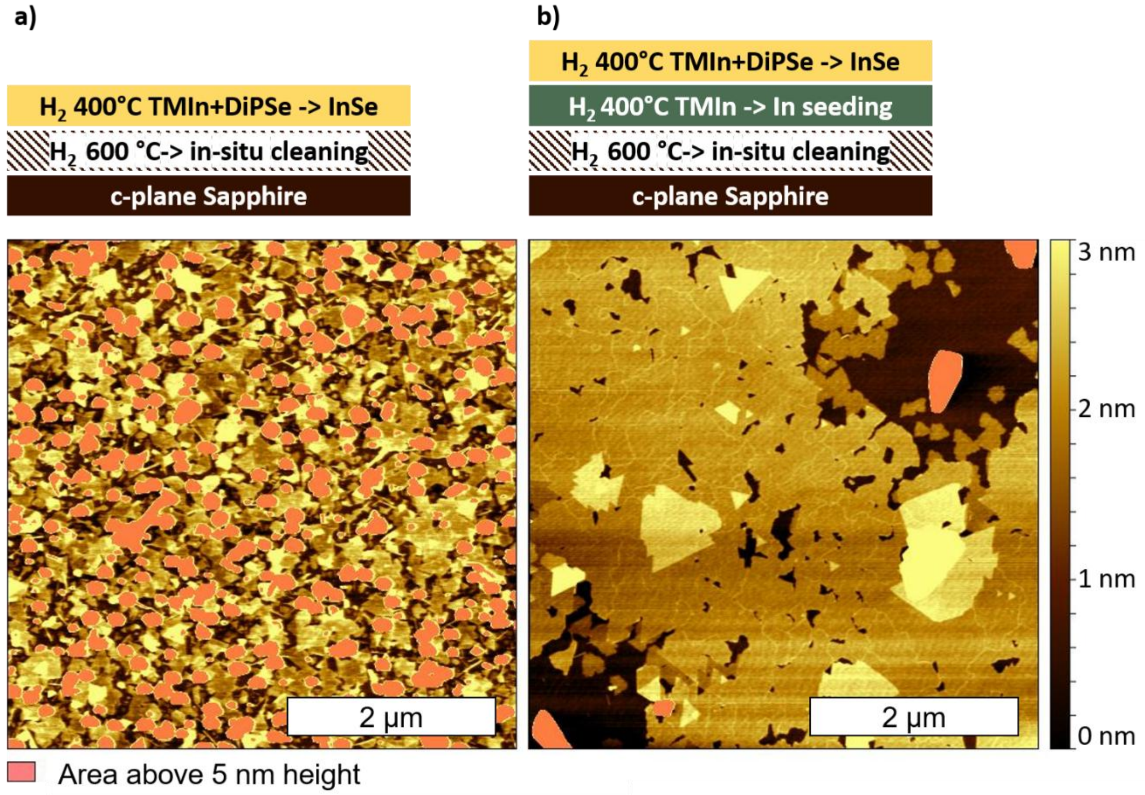

Figure SI3: Influence of nucleation conditions on film morphology: a) AFM image of an InSe layer grown for 3 hours without a preceding In seeding sequence. b) AFM image of an InSe layer grown for 3 hours with a corresponding In-seeding sequence. The respective growth procedure is shown schematically above the two images.

The influence of an indium seeding layer on the growth behavior of InSe is demonstrated through comparative surface morphology analysis. Figure SI3 presents AFM images of two InSe samples grown on c-plane sapphire under otherwise identical conditions, with the only difference being the presence or absence of an indium seeding step.

Figure SI3a shows the sample synthesized without In seeding. It exhibits a high density of surface features exceeding 5 nm in height, indicating rough and discontinuous nucleation. In contrast, Figure SI3b displays the sample grown with a pre-deposited In seeding layer. Here, the surface is significantly smoother and more well-defined, layered domains with lateral sizes approaching 1 µm are clearly observed. These observations indicate that the initial indium seeding step facilitates a more uniform and layered two-dimensional growth. When accounting for the additional indium introduced during this step, the effective Se/In precursor ratio decreases from 900 to approximately 750. Although such an increase in available indium would typically be expected to promote the formation of metallic indium or In_4Se_3 clusters, this was not observed in our case. Instead, the seeded samples exhibit smoother surfaces and larger lateral flake dimensions, suggesting that the indium pre-treatment supports the formation of well-defined 2D InSe layers rather than secondary phases. A similar effect has been reported in the literature, where interlayers—such as Ga based for GaS[5] or W[6] based for WS_2—were found to be critical in enabling epitaxial alignment on sapphire substrates.

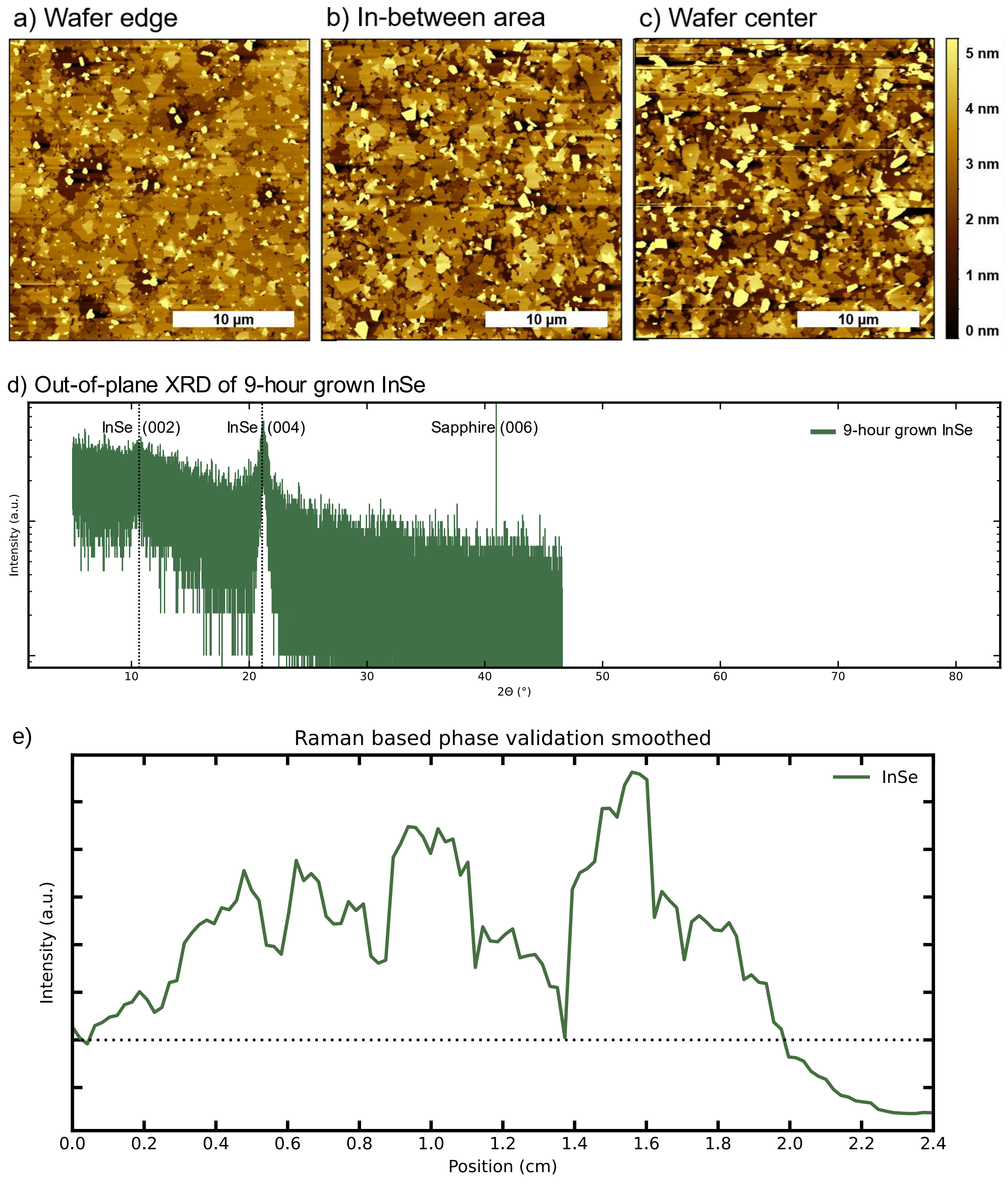

Figure SI4: **Morphology on wafer scale:** From left a) to right c), three AFM measurements of the thick sample are shown. The height and flake sizes of the 2nd, 3rd and subsequent layers are generally in a similar range. Qualitatively, however, it appears that there are fewer tall but relatively larger and interconnected flakes near the wafer edge, while many tall flakes are observed in the central region. d) Out-of-plane 2θ–ω XRD scan of the InSe film on sapphire (green). The diffraction peaks corresponding to the InSe (002) and (004) lattice planes are indicated by black dashed lines, along with the sapphire (006) reflection for reference. (e) Raman-based phase verification of the 9-hour sample was performed across the entire wafer, from the center (0 cm) to the edge (2.4 cm). The plotted intensity

corresponds to the peak area of the InSe $A'_1(1)$ Raman mode, which was fitted using a Lorentzian function and smoothed using a moving-mean filter. The dotted line marks the normalized reference level of 1. A noticeable decrease in the InSe signal is observed near the wafer edge (~2 cm).

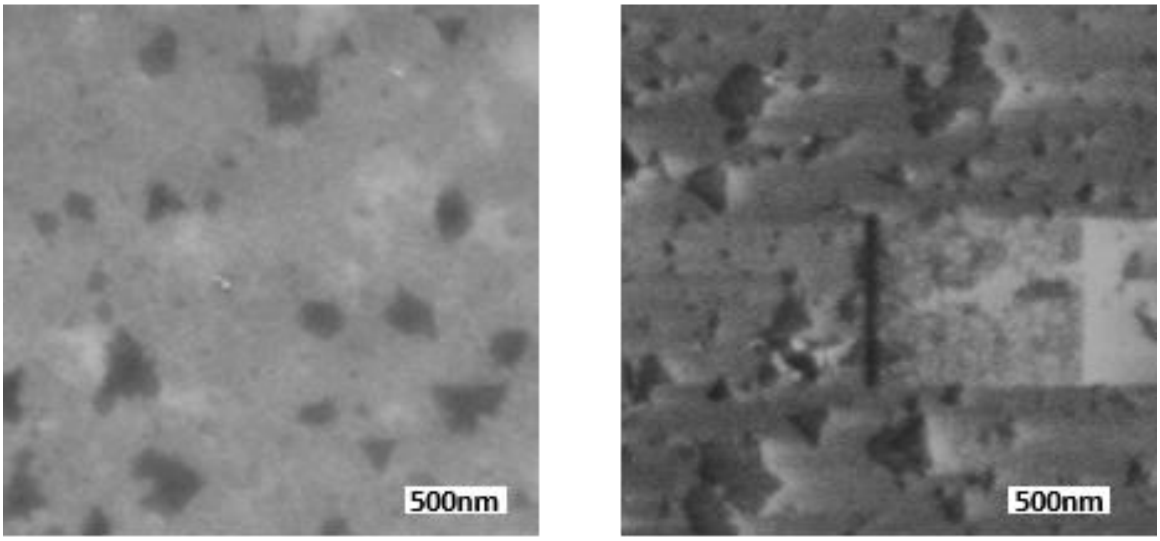

Figure SI5: Beam damage during SEM: Uncoated InSe can be shown to be sensitive to moderate to low electron exposure (2kV, 13pA). For FIB preparation we carried out protective C coating in order to mitigate this effect.

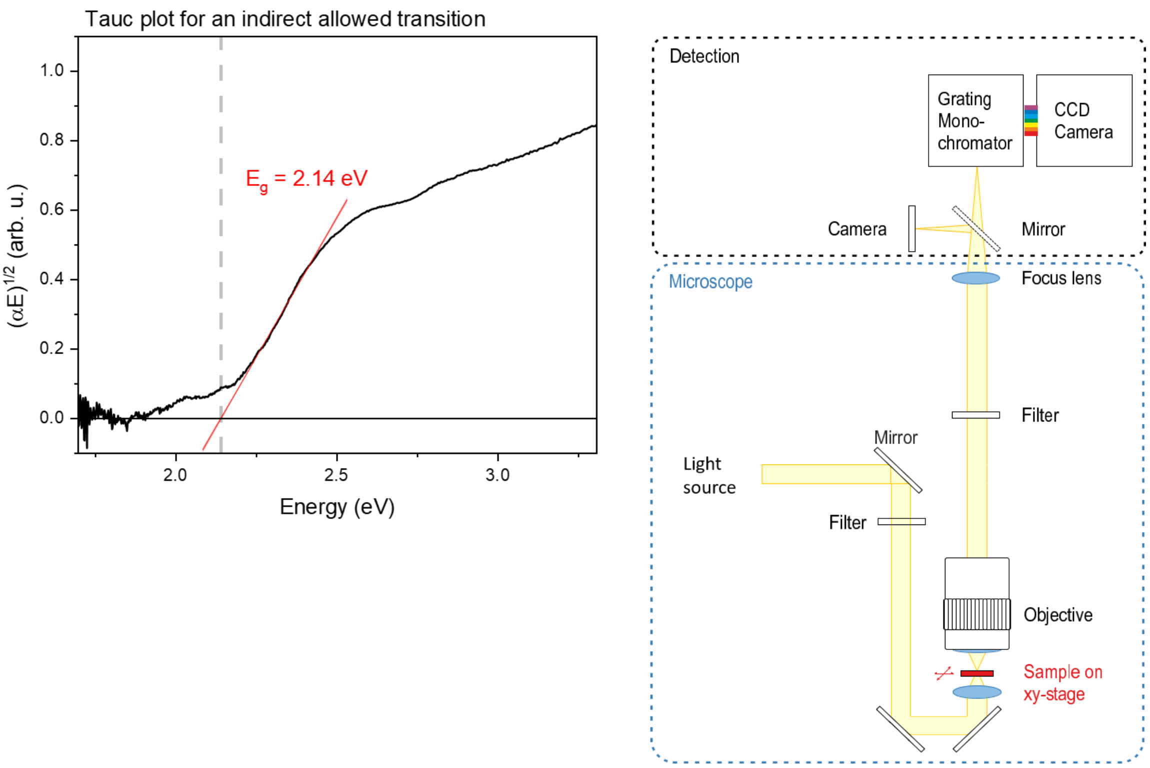

Figure SI6: Microscopic absorbance measurement in transmission geometry: A Tungsten light source is focused on the sample and transmitted light collected from an area with a diameter of approximately 1400 µm using an objective (NA 0.15). The recorded spectrum (on the left) was normed (0 to 1). The band gap onset is evaluated by a Tauc plot at Eg = 2.14 eV. The utilized setup is depicted on the right

The integration of 2D materials into device architectures presents significant challenges due to complex interfaces and the necessity for precise optimization [7]. Additionally, characterizing InSe is particularly challenging due to its susceptibility to decomposition in ambient conditions over time and its sensitivity to electron irradiation (cf. Figure SI5), which can lead to degradation of its properties[8]. To avoid loss of mobility caused by unwanted damage during further device preparation or electron beam-based analysis methods, in this study we measure the carrier mobility of the as-grown samples using THz transmission spectroscopy. This advanced, non-destructive technique allows precise mobility assessment in thin film materials as demonstrated for example for graphene[9] and will provide critical insight into the electronic properties of InSe, guiding further optimization for effective device integration.

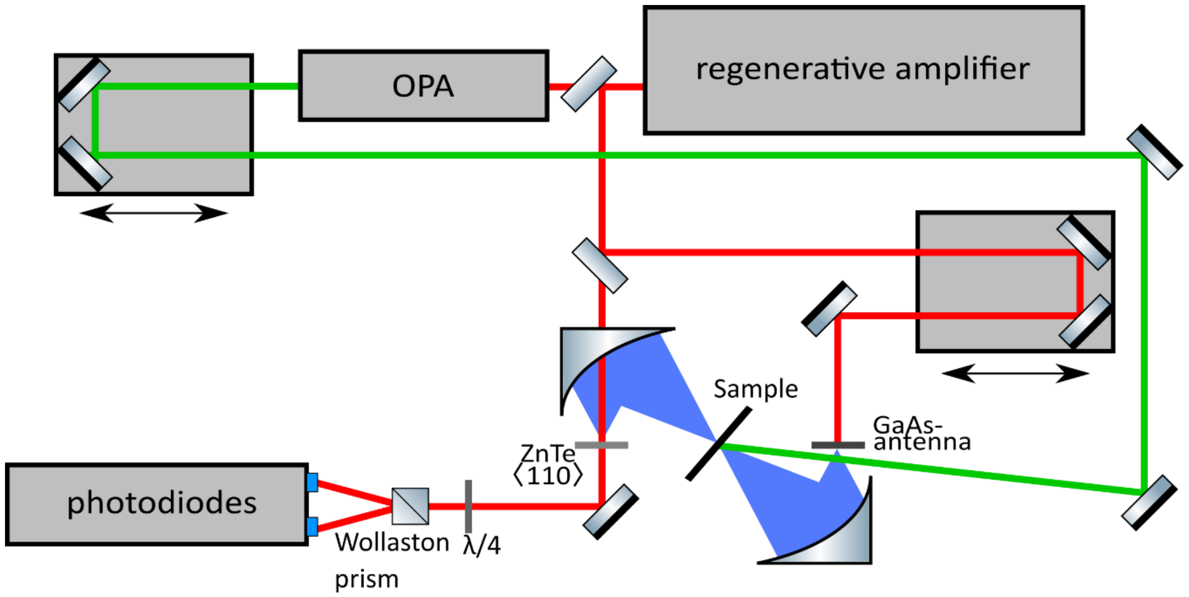

Figure SI7: Schematic of the optical pump–terahertz probe setup: The regenerative amplifier serves as the light source. The green beam path is used for optically exciting the sample, while part of the red beam is directed to a low-temperature-grown GaAs antenna for THz generation. The remaining part of the red beam is used for detecting the THz pulse via electro-optic sampling. A mechanical delay stage allows for precise temporal control between the optical pump and the THz probe pulse.

REFERENCES

[1] S.-J. Baek, A. Park, Y.-J. Ahn, J. Choo, *The Analyst* **2015**, *140*, 250.

[2] C. A. Voigt, M. Tian, R. Peacock, B. K. Wagner, E. M. Vogel, *Journal of Applied Physics* **2024**, *135*.

[3] V. I. Mikla, J. M. Turovci, V. V. Mikla, N. Mehta, *Progress in Solid State Chemistry* **2018**, *49*, 1.

[4] C. Julien, A. Khelfa, N. Benramdane, J. P. Guesdon, *Materials Science and Engineering: B* **1994**, *27*, 53.

[5] O. Maßmeyer, R. Günkel, J. Glowatzki, P. Klement, B. Ojaghi Dogahe, S. R. Kachel, F. Gruber, M. Müller, M. Fey, J. Schörmann et al., *Small (Weinheim an der Bergstrasse, Germany)* **2024**, *20*, e2402155.

[6] A. Cohen, P. K. Mohapatra, S. Hettler, A. Patsha, K. V. L. V. Narayanachari, P. Shekhter, J. Cavin, J. M. Rondinelli, M. Bedzyk, O. Dieguez et al., *ACS Nano* **2023**, *17*, 5399.

[7] a) M. Shrivastava, V. Ramgopal Rao, *Nano letters* **2021**, *21*, 6359; b) L. Jia, J. Wu, Y. Zhang, Y. Qu, B. Jia, Z. Chen, D. J. Moss, *Small Methods* **2022**, *6*, e2101435; c) T. Tan, X. Jiang, C. Wang, B. Yao, H. Zhang, *Advanced Science (Weinheim, Baden-Wurttemberg, Germany)* **2020**, *7*, 2000058; d) J. Jiang, K. Parto, W. Cao, K. Banerjee, *IEEE J. Electron Devices Soc.* **2019**, *7*, 878.

[8] a) H.-W. Yang, H.-F. Hsieh, R.-S. Chen, C.-H. Ho, K.-Y. Lee, L.-C. Chao, *ACS Applied Materials & Interfaces* **2018**, *10*, 5740; b) S. Song, S. Jeon, M. Rahaman, J. Lynch, D. Rhee, P. Kumar, S. Chakravarthi, G. Kim, X. Du, E. W. Blanton et al., *Matter* **2023**, *6*, 3483; c) E. Sim, D. Kim, T. H. Nguyen, J. Kim, H. E. Cho, S. Koo, S. Cho, J. H. Shim, S. C. Lim, K. Ihm, *ACS Applied Materials & Interfaces* **2024**, *16*, 65628.

[9] C. Lee, J. Y. Kim, S. Bae, K. S. Kim, B. H. Hong, E. J. Choi, *Applied Physics Letters* **2011**, *98*.
